# Fronto‐Orbital Osteoblastoma With Unilateral Exophthalmos: A Rare Presentation and Review of Diagnostic Challenges

**DOI:** 10.1002/ccr3.70073

**Published:** 2025-01-06

**Authors:** Mohammadhossein Khorraminejad‐Shirazi, Ali Nabavizadeh, Anita Bojnoordi, Dena Firouzabadi, Abbas Rakhsha, Seyed Ali Hosseini, Amirreza Dehghanian

**Affiliations:** ^1^ Department of Pathology, School of Medicine Jahrom University of Medical Sciences Jahrom Iran; ^2^ Department of Pathology, School of Medicine Shiraz University of Medical Sciences Shiraz Iran; ^3^ Student Research Committee Shiraz University of Medical Sciences Shiraz Iran; ^4^ Otolaryngology Research Center, Department of Otolaryngology Shiraz University of Medical Sciences Shiraz Iran; ^5^ Clinical Pharmacy Department, Shiraz School of Pharmacy Shiraz University of Medical Sciences Shiraz Iran; ^6^ Shahid Faghihi Hospital, Clinical Pharmacy Department Shiraz University of Medical Sciences Shiraz Iran; ^7^ Department of Neurosurgery, School of Medicine Shiraz University of Medical Sciences Shiraz Iran; ^8^ Trauma Research Center Shiraz University of Medical Sciences Shiraz Iran; ^9^ Molecular Pathology and Cytogenetics Division, Department of Pathology Shiraz University of Medical Sciences Shiraz Iran

**Keywords:** case report, craniofacial, fronto‐orbital region, osteoblastoma

## Abstract

Osteoblastoma is an uncommon benign bone tumor rarely involving the craniofacial skeleton. Manifestations in the fronto‐orbital region are exceptionally rare. A 19‐year‐old man presented with persistent headache, nausea, vomiting, right eye pain, and longstanding right exophthalmos. Imaging revealed a heterogeneous enhancing lesion located in the right anterior cranial fossa‐orbital apex junction causing pressure on the orbital roof. Differential diagnoses included fibrous dysplasia and meningioma. The tumor was resected via frontal craniotomy. Definitive diagnosis of osteoblastoma was achieved postoperatively through histopathological examination and IHC studies. Craniofacial osteoblastomas, especially in the fronto‐orbital region, are exceptionally rare and may present radiological features similar to other bone pathologies. Accurate diagnosis hinges on histopathological evaluation. A multidisciplinary approach is pivotal for successful diagnosis and treatment of such challenging cases.

**Abbreviations**
CT: computed tomography
H&E: hematoxylin and eosin

**Summary**

- Craniofacial osteoblastomas are exceptionally rare and may present radiological features similar to other bone pathologies.
- This underscores the crucial role of histopathology in arriving at a definitive diagnosis and guiding appropriate surgical management.

## 1 Introduction

Osteoblastoma is a benign, bone‐forming tumor characterized by its slow growth. Although relatively rare, comprising < 1% of primary bone tumors, their manifestation in the craniofacial region, specifically the fronto‐orbital bones, is exceptionally rare [1, 2, 3], with fewer than five reports [4, 5, 6, 7]. The complex anatomy of the fronto‐orbital region presents diagnostic and therapeutic challenges in managing these uncommon tumors [8]. The differential diagnosis for fronto‐orbital lesions is broad, including osteosarcoma, fibrous dysplasia, and other bone tumors which can mimic osteoblastomas clinically, radiographically, and histologically [9]. This makes definitive diagnosis difficult prior to surgical resection.

Here, we present a rare case of fronto‐orbital osteoblastoma with orbital involvement, presenting with proptosis, making this a descriptive case that demands heightened attention to possible differential diagnoses and radiological features.

## 2 Case History/Examination

A 19‐year‐old man presented to the neurosurgery department at Chamran Hospital, Shiraz, Iran with a 3‐month history of persistent headache, nausea, vomiting, and right eye pain. Notably, the patient also complained about forward displacement of his right eye that had been present since birth. His medical history was unremarkable, without any skeletal diseases, history of trauma, and relevant family history.

Physical examination revealed no signs of inflammation, erythema, or palpable masses around the right orbit. Also, systemic physical examination showed no unusual findings. Notable right‐sided proptosis was present, though the globe itself was without additional abnormalities on external inspection. Ophthalmologic evaluation indicated intact visual acuity and brisk pupillary reflexes bilaterally. Routine laboratory tests were within normal limits.

**FIGURE 1:**
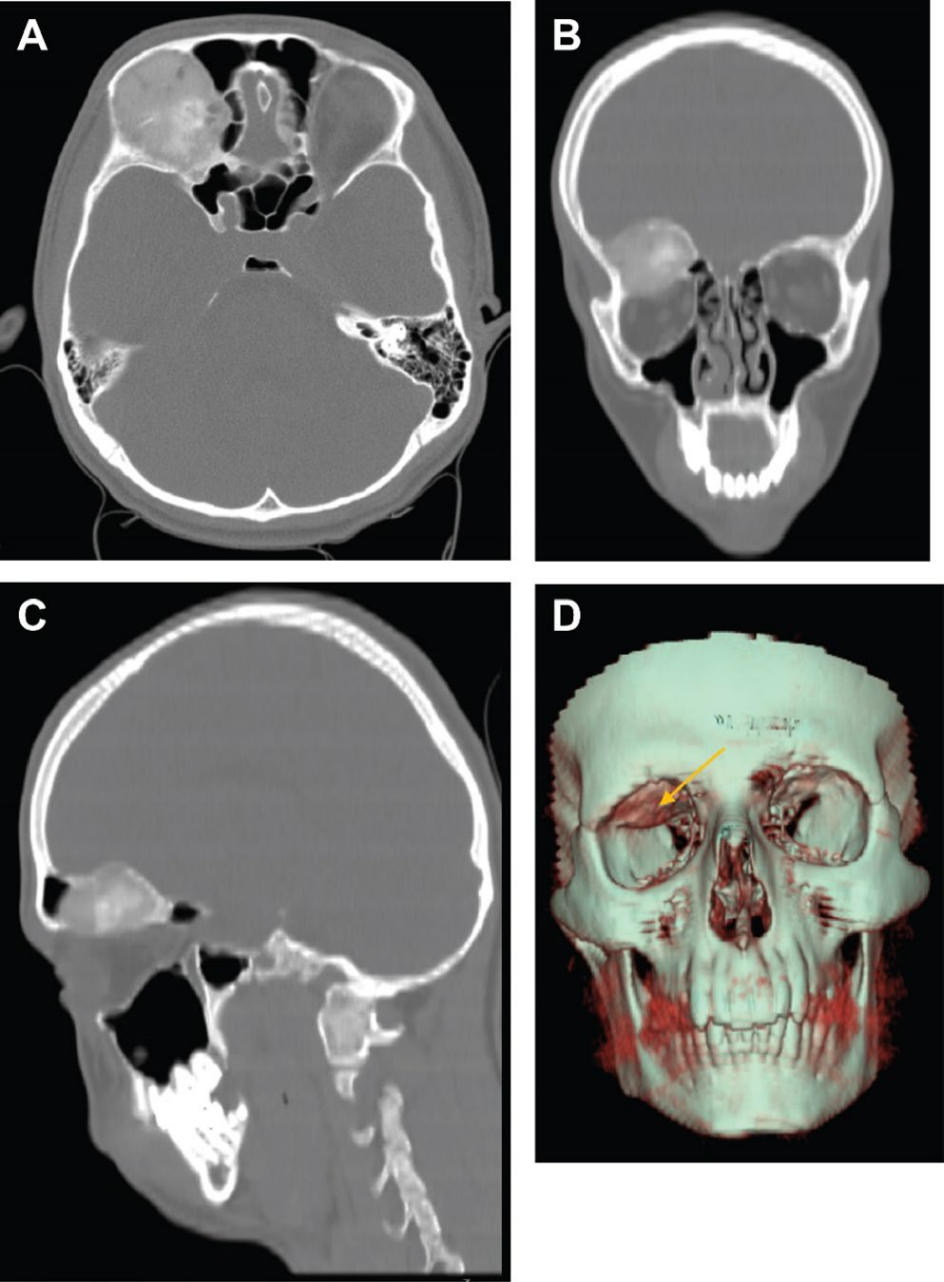
CT scans of the bony lesion. (A) Axial section displaying ground glass matrix changes of the osteoblastoma. (B) Coronal view showing the expansile bony lesion centered at the anterior cranial base and extending to involve the orbital roof. (C) Sagittal reconstruction depicting the extra‐axial lesion. (D) 3D reconstructed CT image showing expansion of the bone resulting in a decrease in the volume of the right orbit.

**FIGURE 2:**
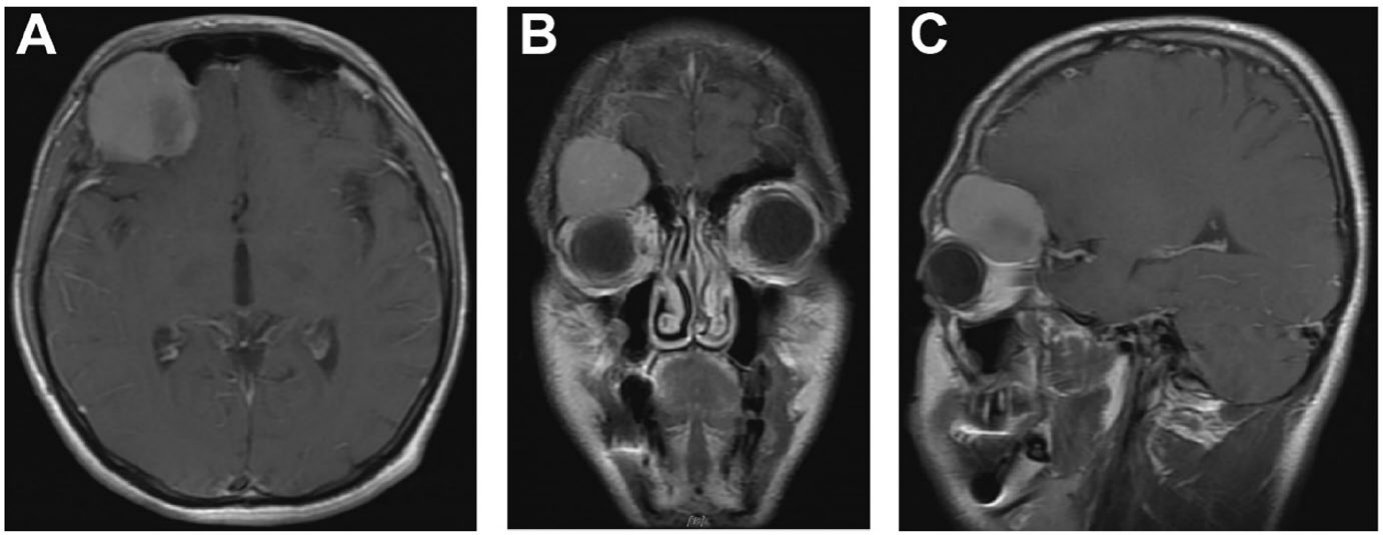
MRI characteristics of the fronto‐orbital osteoblastoma. (A) Axial postcontrast T1‐weighted image depicting the tumor exerting pressure on the right orbital roof. (B) Coronal T2‐weighted image revealing a homogenously hyperintense extra‐axial mass centered at the anterior cranial fossa‐orbital apex junction with inferior displacement of the globe. (C) Postcontrast sagittal T1‐weighted image showing vivid enhancement of the lesion.

Computed tomography (CT) of the orbit and skull revealed a 43 × 30 mm bony lesion located at the roof of the right orbit, which had led to an expansion of the bone (Figure 1). This expansion consequently resulted in a decrease in the volume of the right orbit, exerting a pressure effect on the superior rectus muscle and causing proptosis of the right globe. Radiographic features showed ground glass matrix morphology, raising consideration of fibrous dysplasia as a differential diagnosis. Subsequent magnetic resonance imaging (MRI) defined an extra‐axial, homogenously enhancing 41 × 40 × 30 mm lesion centered at the right anterior cranial fossa‐orbital apex junction, exerting additional pressure on the orbital roof with associated exophthalmos (Figure 2). These findings were consistent with meningioma as a potential differential diagnosis.

## 3 Methods

Gross total excision of the frontal bone lesion was performed, extending up to the point where the ethmoid sinus was visible. A tissue sample from the mass was sent for pathological examination. Following the removal of the lesion, the base of the skull was repaired using subcutaneous fat harvested from the patient's thigh.

**FIGURE 3:**
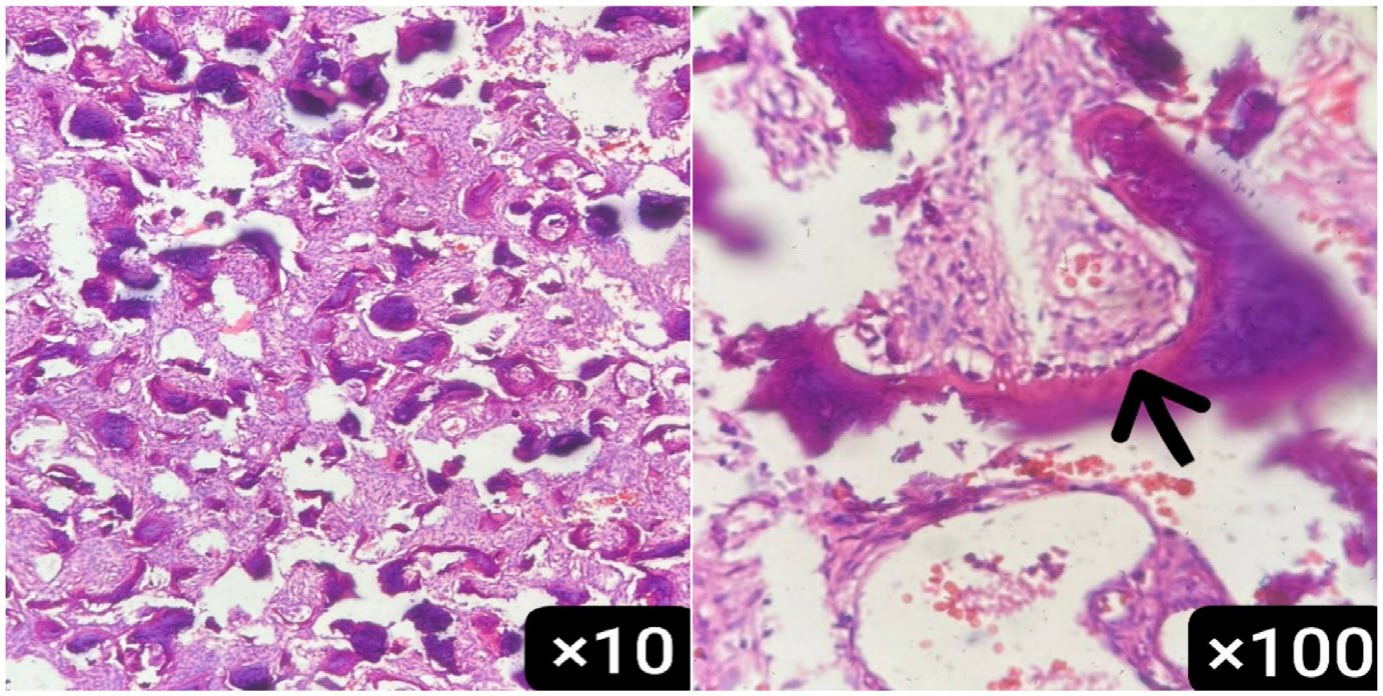
Histopathologic examination showed bony trabeculae rimmed by osteoblasts.

**FIGURE 4:**
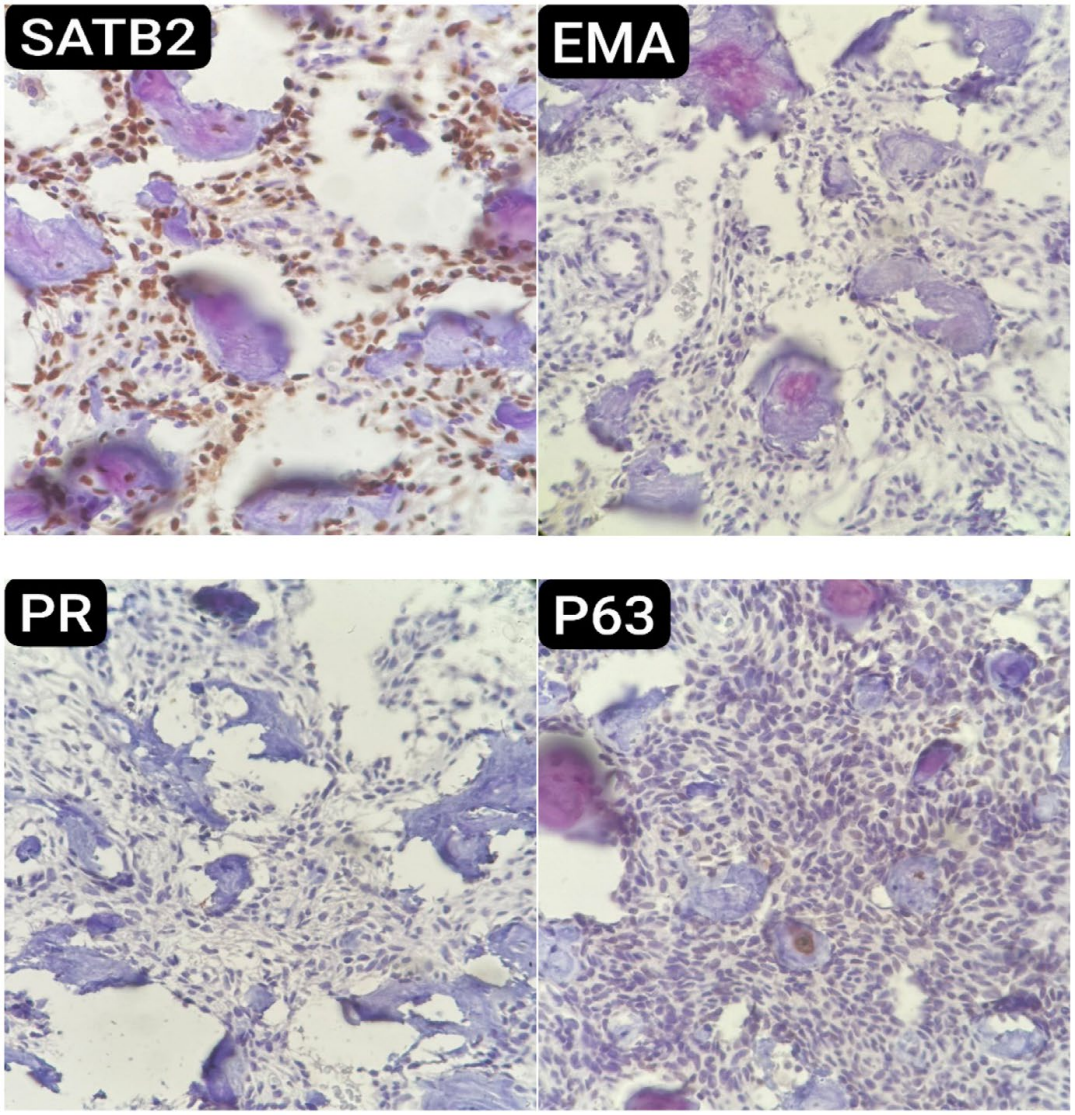
Immunohistochemical staining of the specimen shows positive staining for SATB2, consistent with osteoblastoma, and negative staining for EMA, PR, and P63.

Histopathological examination of the resected specimens revealed anastomosing trabeculae of woven bone, lined by single layer of osteoblasts, separated by fibrovascular stroma and some extravasated erythrocytes (Figure 3). Immunohistochemistry (IHC) study of the specimen was positive for SATB2 and showed negative staining for EMA, PR, and P63. These findings were consistent with the diagnosis of osteoblastoma (Figure 4).

## 4 Conclusion and Results

The postoperative course was uncomplicated, with the patient experiencing marked improvement in his preoperative symptoms of headache, ocular discomfort, and exophthalmos. Postoperative CT imaging confirmed complete removal of the bony expansion, with no evidence of residual tumor.

## 5 Discussion

We present a rare case of osteoblastoma originating from the orbital roof that clinically manifested with unilateral exophthalmos. Although osteoblastomas represent < 1% of all primary bone tumors, their occurrence within the craniofacial bones is exceptionally uncommon, accounting for only 15% of osteoblastoma cases [10]. Among craniofacial sites, the mandible is most frequently involved, comprising 14%–30% of cases, followed by the maxilla (8%–15%), with the calvarial bones and orbital region least commonly affected [11].

The clinical presentation of craniofacial osteoblastomas is often nonspecific, with local pain being the most common symptom, reported in 75%–90% of cases. Less frequent manifestations include swelling, facial asymmetry, trismus, vision changes, and cranial nerve deficits [12]. Notably, exophthalmos as a presenting sign, as seen in our patient, is distinctly rare, further highlighting the uniqueness of this case [13].

Radiographically, osteoblastomas exhibit diverse and nonspecific features that can mimic numerous other entities, including fibrous dysplasia, ossifying fibroma, meningioma, and osteosarcoma [7]. Osteoblastoma typically appears on imaging as a well‐defined lytic lesion with a central nidus, often surrounded by a thin sclerotic rim, and can display vivid enhancement on MRI due to its vascularity, making modalities like radiographs, CT, and MRI integral for its diagnosis and differentiation from other bone lesions. However, significant overlap exists with other bone pathologies, necessitating histopathological confirmation for a definitive diagnosis [14, 15]. In our case, the initial differential diagnoses based on CT and MRI findings included fibrous dysplasia and meningioma. Definitive diagnosis was only possible after surgical resection and pathological examination.

For the treatment the osteoblastoma was completely resected via frontal craniotomy. Other successful techniques described for orbital roof osteoblastomas include pre‐septal orbital and endoscopic approaches [5]. However, complete excision remains the goal regardless of surgical approach, as subtotal resection confers a higher risk of recurrence given the high risk of recurrence [16].

In summary, osteoblastomas are uncommon primary bone tumors that infrequently involve the craniofacial bones and orbital region. Our case showcased a rare presentation of fronto‐orbital osteoblastoma manifesting with unilateral exophthalmos. Although imaging modalities are useful for characterization, significant radiographic overlap exists in the differential diagnosis. This underscores the crucial role of histopathology in arriving at a definitive diagnosis and guiding appropriate surgical management. Clinicians should be cognizant of osteoblastoma as a potential etiology of craniofacial and orbital masses. A high index of suspicion and multidisciplinary approach are paramount for timely diagnosis and treatment of this rare entity.

## Author Contributions

**Mohammadhossein Khorraminejad‐Shirazi:** writing – original draft, writing – review and editing. **Ali Nabavizadeh:** writing – original draft, writing – review and editing. **Anita Bojnoordi:** writing – original draft, writing – review and editing. **Dena Firouzabadi:** writing – review and editing. **Abbas Rakhsha:** writing – review and editing. **Seyed Ali Hosseini:** writing – original draft, writing – review and editing. **Amirreza Dehghanian:** writing – original draft, writing – review and editing.

## Ethics Statement

The present study was approved by the Medical Ethics Committee of Shiraz University of Medical Sciences (#29593). The purpose of this report was completely explained to the patient and written inform consent was obtained from the patient.

## Consent

Written informed consent for publication of the patient's clinical details and pathologic images was obtained from the patient. A copy of the consent form is available for review by the Editor of this journal.

## Conflicts of Interest

The authors declare no conflicts of interest.

## Data Availability

Data of the patient can be requested from authors. Please write to the corresponding author if you are interested in such data.
